# Repurposing povidone-iodine to reduce the risk of SARS-CoV-2 infection and transmission: a narrative review

**DOI:** 10.1080/07853890.2022.2076902

**Published:** 2022-05-20

**Authors:** Nicole-Ann Lim, Ooiean Teng, Chester Yan Hao Ng, Lena X. Y. Bao, Paul Anantharajah Tambyah, Amy M. L. Quek, Raymond C. S. Seet

**Affiliations:** a Department of Medicine, Yong Loo Lin School of Medicine, National University of Singapore, Singapore, Singapore; b Healthy Longevity Translational Research Program, Yong Loo Lin School of Medicine, National University of Singapore, Singapore, Singapore

**Keywords:** Povidone-iodine, mechanisms, SARS-CoV-2, clinical studies, adverse effects

## Abstract

**Background:**

Accumulating data suggest antiviral effects of povidone-iodine against the Severe acute respiratory syndrome coronavirus 2 (SARS-CoV-2) virus. This narrative review aims to examine the antiviral mechanisms of povidone-iodine, efficacy of povidone-iodine against the SARS-CoV-2 virus, and safety of povidone-iodine to human epithelial cells and thyroid function.

**Methods:**

We searched the electronic databases PubMed, Embase, Cochrane Library, ClinicalTrials.gov and World Health Organization’s International Clinical Trials Registry Platform for articles containing the keywords “povidone-iodine”, “SARS-CoV-2” and “COVID-19” from database inception till 3 June 2021.

**Results:**

Despite *in vitro* data supporting the anti-SARS-CoV-2 effects of povidone-iodine, findings from clinical studies revealed differences in treatment response depending on study settings (healthy *vs.* hospitalized individuals), treatment target (nasal *vs.* oral *vs.* pharynx), method of administration (oral rinse *vs.* gargle *vs.* throat spray) and choice of samples used to measure study endpoints (nasopharyngeal *vs.* saliva). One large-scale clinical trial demonstrated reduction in the incidence of SARS-CoV-2 infection among participants who administered povidone-iodine 3 times daily during an active outbreak. Povidone-iodine is also used to disinfect the oro-pharyngeal space prior to dental or otolaryngology procedures. Although existing data suggest minimal impact of povidone-iodine on thyroid function, high-quality safety data are presently lacking.

**Conclusions:**

Povidone-iodine application to the oropharyngeal space could complement existing non-pharmacological interventions to reduce SARS-CoV-2 infection especially in high exposure settings.

**Key messages**
- Accumulating data suggest antiviral effects of povidone-iodine against the SARS-CoV-2 virus.
- Findings from clinical studies reveal differences in treatment response depending on study settings, treatment target, method of administration and choice of samples used to measure study endpoints. One large-scale clinical trial observed reduction in the incidence of SARS-CoV-2 infection among participants who administered povidone-iodine 3 times daily during an active outbreak.
- Povidone-iodine application to the oropharyngeal space could complement existing non-pharmacological interventions to reduce SARS-CoV-2 infection especially in high exposure settings.

## Introduction

Povidone-iodine is a broad-spectrum antiseptic that is widely used to disinfect surgical sites and in wound management [1]. By targeting cell membranes, povidone-iodine exerts potent anti-bacterial and anti-fungal effects [2], and is generally well tolerated when applied to skin surfaces [3]. Interest in povidone-iodine has surged during this pandemic following reports from preclinical and clinical studies supporting its antiviral effects against the severe acute respiratory syndrome coronavirus 2 (SARS-CoV-2) virus [4–6], in addition to other respiratory viruses such as influenza [7], SARS-CoV [8] and MERS-CoV [8–10]. In cell culture studies, povidone-iodine inhibits SARS-CoV-2 replication rapidly [4,6], prompting clinical studies to explore the use of povidone-iodine to disinfect the oropharyngeal space before dental [11] and otolaryngology [12] procedures. When applied regularly to the oropharyngeal space, one large-scale clinical trial that was conducted during an active outbreak showed povidone-iodine could significantly reduce SARS-CoV-2 infection by more than 20% compared with an active comparator among residents living in a crowded dormitory [13].

Previous reviews have focussed on the use of povidone-iodine to disinfect the oral cavity during dental [11,14–18] and otolaryngology procedures [12,19,20] but did not elaborate on its practical application in endemic and high-transmission settings in the community. There is an emergent need to evaluate povidone-iodine in this context, in light of accumulating data surrounding its ability to manage transmission risks in the community. This review aims to examine the pharmacology of povidone-iodine and review data from preclinical and human studies to critically appraise the antiviral effects of povidone-iodine against the SARS-CoV-2 virus. We also reviewed the literature to examine safety of povidone-iodine when applied locally to the oropharyngeal space and its effects to thyroid function.

## Materials and methods

This narrative review was based on a literature search of electronic databases PubMed, Embase, The Cochrane Library, ClinicalTrials.gov and World Health Organization’s International Clinical Trials Registry Platform, from database inception up till 3 June 2021. The grey literature was also searched, including Google Scholar, bioRxiv and medRxiv. The search strategy included the terms “povidone-iodine”, “SARS-CoV-2” and “COVID-19”. The full inclusion and exclusion criteria are appended in Supplementary Table 1.

### Pharmacology and antiviral actions of povidone-iodine

**Figure 1.**
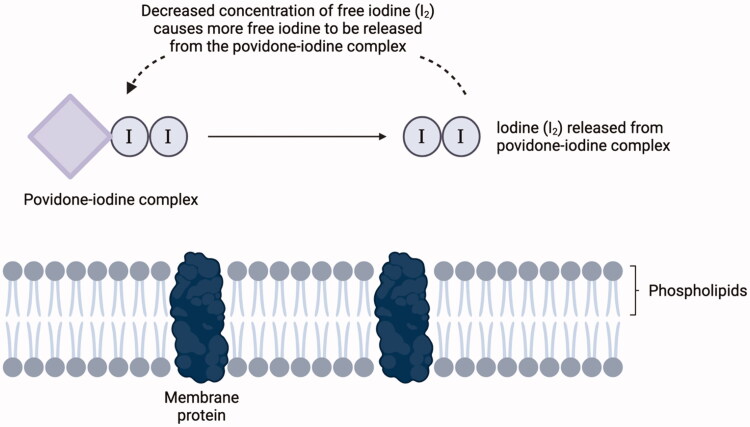
Effect of povidone-iodine on pathogen cellular structures. In aqueous solution, povidone-iodine dissolves to release free iodine, which then oxidizes membrane proteins on the surfaces of virus and bacterial pathogens [21].

**Figure 2.**
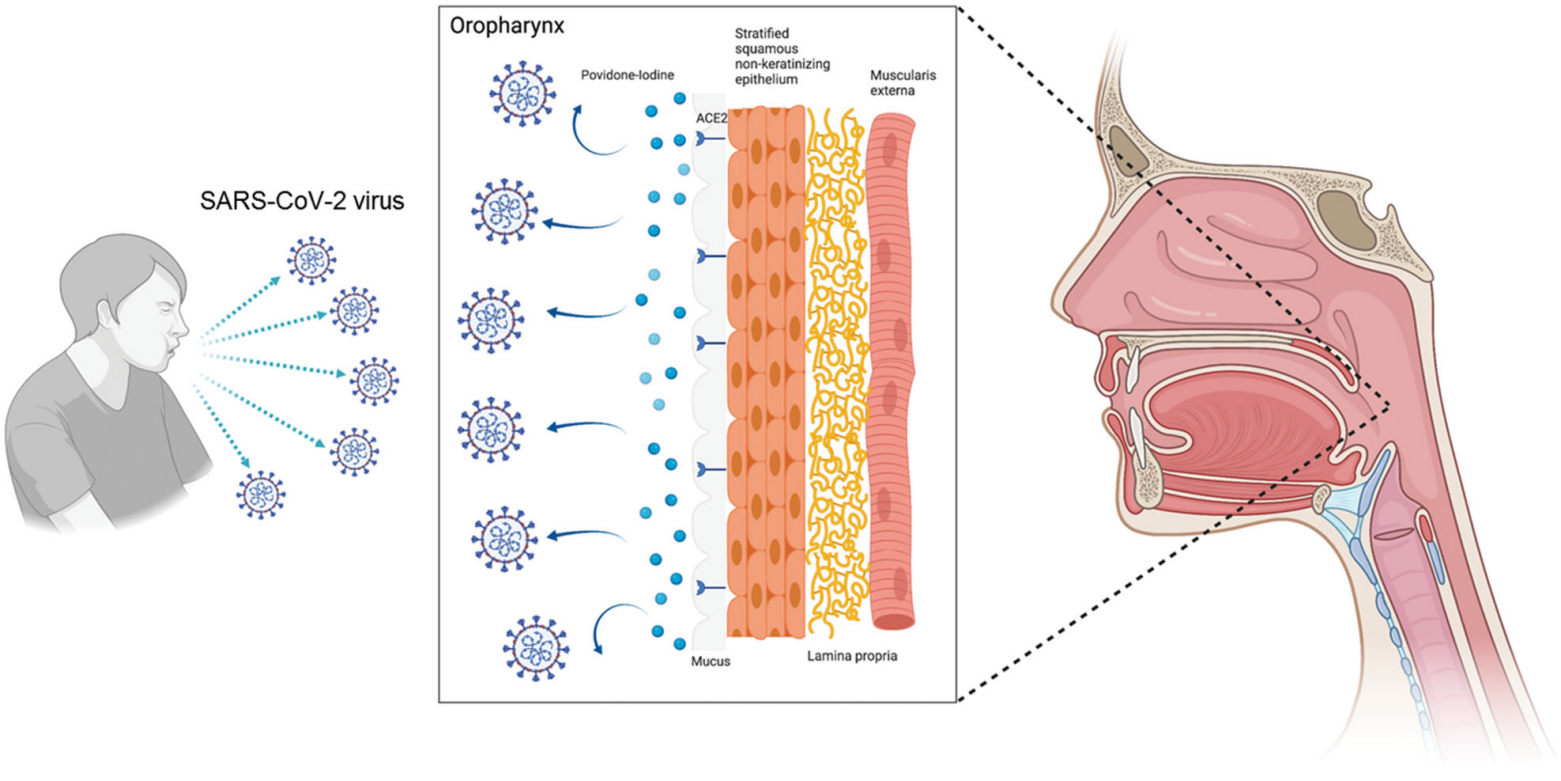
Povidone-iodine exerting its local anti-SARS-CoV-2 effects. The oropharynx is lined by stratified squamous non-keratinizing epithelium, with its underlying lamina propria and muscularis externa [98]. These epithelial cells contain ACE2 receptors, which are entry points for SARS-CoV-2 into humans tissues [90]. By coating the oropharynx, povidone-iodine blocks entry of the SARS-CoV-2 virus into host cells and prevents its spread to adjacent tissues.

Povidone-iodine is a water-soluble complex of iodine and its carrier molecule polyvinylpyrrolidone (PVP, also called povidone) [21]. Povidone-iodine maintains an equilibrium state in an aqueous solution and exerts its antimicrobial effects after free iodine dissociates from PVP [21]. The free iodine (I_2_) is presumed to kill bacteria and viruses by oxidizing membrane proteins on the surfaces of bacteria and viruses (Figure 1) [22]. Once I_2_ is consumed [2], other iodine species in the solution (e.g. I_3_^−^ , I^+^ and IO_3_^−^) are then used to replenish the decreased levels of I_2_ [23]. In histology studies, povidone-iodine has been shown to spread parallel to the external surface of the epithelium, penetrating to the level of the prickle cells of the stratum spinosum [24]. As blood supply to the mucosa is located deeper in the epidermis, direct absorption of povidone-iodine into the circulation is limited (Figure 2) [24]. The antimicrobial efficacy of povidone-iodine follows a bell-shaped curve where optimal efficacy occurs between 0.1% and 1.0% concentration, thereby allowing reduced amounts of iodine to exert its antiseptic effects [23]. Povidone-iodine interacts with bacterial and fungal cell walls and causes pore formation or solid–liquid interfaces at the lipid membrane, resulting in loss of cytosol materials and enzyme denaturation in cell walls [2]. In clinical practice, povidone-iodine is widely used to prevent surgical site infection, and in treatment of burns, wounds and eye infections [22–29]. The antiviral mechanisms of povidone-iodine are less understood [1]. One study suggested that povidone-iodine could reduce replication of the avian and human influenza A by inhibiting the N1, N2 and N3 neuraminidases and viral haemagglutinin [7]. Other studies hypothesized that povidone-iodine could increase release of reactive oxygen species intermediaries capable of triggering oxidative damage to key virus structures [2,30].

Following entry of the SARS-CoV-2 virus into upper respiratory epithelial cells, further replication of the virus occurs, resulting in infection and spread to adjacent tissues [31]. Virus particles present on the surface of cells could also migrate to more distant respiratory tissues through the continuous actions of cilia on epithelial cells and by mixing with saliva and mucous before reaching the bronchi and alveoli [32]. The antiviral effects of povidone-iodine on coronaviruses were first suggested by studies that showed significant reduction in virus load following exposure to the SARS-CoV-1 and MERS-CoV viruses (Supplementary Table 2) [8–10]. Interests in the anti-viral effects of povidone-iodine against SARS-CoV-2 stem from preclinical studies showing significant reduction in virus load following exposure to povidone-iodine in concentrations ranging between 0.45% and 10% (Table 1) [6,12,32,36]. One study attributed the antiviral effects of povidone-iodine to its direct cytotoxicity to infected host cells [41] while another study suggested that povidone-iodine could destabilize the lipid envelope, lyse spike proteins and oxidize the nucleic acid of virus particles [42]. These actions are less likely to be mediated by binding of povidone-iodine to specific viral targets as data from *in silico* studies suggest poor affinity of povidone-iodine for SARS-CoV-2 proteins [43].

**Table 1.** Study characteristics and results for in-vitro and animal studies.

Studies	Type of povidone-iodine	SARS-CoV-2 virus titre reduction	Povidone-iodine formulation and concentration	Shortest contact time taken for reduction in SARS-CoV-2 titres
[4]	Betadine^®^ antiseptic solution (povidone-iodine 10% w/v), Betadine^®^ antiseptic skin cleanser (povidone-iodine 7.5% w/v), Betadine^®^ gargle and mouth wash (povidone-iodine 1.0% w/v) and Betadine^®^ throat spray (povidone-iodine 0.45% w/v)	≥4 log10	0.45% throat spray	30 s
			0.5% gargle/mouth wash 1% gargle/mouth wash	
			7.5% skin cleanser	
			10% antiseptic solution	
Bidra et al., 2020 [5]	Povidone-iodine oral rinse antiseptic solutions (Veloce BioPharma; Fort Lauderdale, FL)	≥3 log10	0.5%, 1.25% and 1.5%	30 s
		≥4 log10	0.5%, 1.25% and 1.5%	15 s
[6]	Oral rinse antiseptic solution (Veloce BioPharma) consisting of various concentrations of aqueous povidone-iodine as the sole active ingredient	≥3 log10	0.5%, 0.75% and 1.5%	30 s
[33]	Povident (Huddersfield Pharmacy Specials), surfactant free	≥4 log10	0.58 %	1 min
[12]	Nasal rinse antiseptic solution (from Veloce BioPharma) that contains aqueous povidone-iodine as the sole active ingredient	≥3 log10	2.5%, 1.25% and 0.50%	15 s
[34]	Betadine^®^ gargle and mouthwash (1% povidone-iodine)	≥5 log10	1%	15 s
			0.5%	30 s
		≥4 log10	0.5%	15 s
[35] [Preprint]	Povidone-iodine mouthwash (Videne)	≥3 log10	0.5%	30 s
[36] [Preprint]	Nasodine Antiseptic Nasal Spray (0.5% povidone-iodine) by Firebrick Pharma Limited, Melbourne Australia.	≥3 log10	0.5% Nasodine	15 s
			0.5% povidone-iodine alone (prepared in saline)	5 min
		≥4 log10	0.5% Nasodine	5 min
			0.5% povidone-iodine alone (prepared in saline)	15 min
[37]	Povidone iodine, available commercially at 2%	≥3 log10	1%	60 s
Liang et al., 2020 [38] [Preprint]	Two sustained release povidone-iodine in-situ gel forming formulations, IVIEW-1201 ophthalmic eye drops and IVIEW-1503 nasal spray, consisting of 1.0 % and 0.6% of Povidone-iodine (w/w), respectively	≥3 log10	0.5% and 0.9% ophthalmic eye drop	30 s
			0.3% and 0.54% nasal spray	
			0.28% ophthalmic eye drop	10 min
			0.17% nasal spray	
[39]	Nasal antiseptic solutions and oral rinse antiseptic solutions consisting of aqueous povidone-iodine (Veloce BioPharma)	≥4 log10	1.0%, 2.5%, 5.0% nasal antiseptic 1.0%, 1.5%, 3.0% oral rinse antiseptic	60 s
[40]	0.5% povidone-iodine oral rinse formulation (Betadine^®^ Oral Rinse, Avrio Health L.P.)	≥4 log10	0.5%	30 s

### Efficacy of povidone-iodine against the SARS-CoV-2 virus

Despite these *in vitro* data, there is paucity of high-quality data from human studies. Several studies have focussed on the application of povidone-iodine to disinfect the oropharyngeal space during dental and otorhinolaryngology procedures, with a majority showing a reduction in virus titres (Table 2). One large-scale clinical trial that was conducted during an active SARS-CoV-2 outbreak within a crowded dormitory showed a significant absolute risk reduction by more than 20% among participants who were randomized to receive povidone-iodine throat spray compared with those who received vitamin C [13]. Other than this preventive trial [13], most studies that examined the efficacy of povidone-iodine enrolled cohorts ranging from 4 to 189 participants [48,52]. Some of these studies measured virus load from nasopharyngeal samples [13,44,47,49], while others measured virus load in saliva [48,45,51]. As few studies correlated their cycle threshold (Ct) values with virus load, data from existing studies cannot be pooled to reliably compare the overall effects of povidone-iodine on virus load. Existing PCR methods do not distinguish between inactivated “dead” and “viable” viruses which are necessary to assess the efficacy of povidone-iodine. For example, although two clinical studies observed significant reduction in Ct values following povidone-iodine mouth rinse that is comparable to levels following chlorhexidine use [45,46], another study did not observe statistically significant differences in pre-post levels [51]. Data from recovered COVID-19 patients indicate that PCR positivity could occasionally persist for more than 5 months [53] in the absence of infectivity as verified using Vero-E6 cell cultures [54]. Despite reports of rapid inactivation of the SARS-CoV-2 virus in the laboratory [4,34], Seneviratne and colleagues reported that reduction in virus load only occurs 6 h after povidone-iodine mouth wash use in humans; the lag-time may be due to the initial salivary washout of povidone-iodine and its delayed downstream actions [51]. Future adequately powered studies should consider examining the amount and viability of the virus from the nasal and oropharyngeal mucosa, and standardizing the methods and timing of sample collection following povidone-iodine use.

**Table 2.** Clinical studies examining povidone-iodine in SARS-CoV-2 infected patients.

Study	Demographic details			Presence of control (Y/N)	Type of intervention	Type of sample	Quantification method	Conclusions
	Sample size	Age	Gender (male)					
[44]	Intervention 135 Control 54	43.98 ± 12.67	Intervention 118 Control 41	Y	Nasal irrigation Nasal spray	Nasopharyngeal	Virus clearance on RT-PCR	Nasopharyngeal clearance with all strengths of povidone-iodine nasal irrigation and spray
[45]	Intervention 27 Control 9	45.3 ± 16.7	Intervention 13 Control 2	Y	Oral gargle	Salivary	Ct values on RT-PCR	Reduction in virus load with 1% povidone-iodine compared with distilled water wash.
[46] [Preprint]	Intervention 15 Control 13	Between 44 and 45	Intervention 9 Control 10	Y	Oral mouth rinse	Salivary	Virus clearance on RT-PCR	None of the mouthwashes reduced virus titres compared with baseline levels
[47]	Intervention 12 Control 12	Intervention 33 (23–46) Control 57 (45–68)	Intervention 4 Control 4	Y	Oral mouthwash and gargle Nasal pulverization using an intranasal mucosal atomization device Dab of ointment on nasal mucosa	Nasopharyngeal	Virus clearance on RT-PCR	Greater reduction in virus titres following exposure to povidone-iodine compared with controls (75% *vs.* 32% reduction).
[48]	Intervention 4	61	2	N	Oral rinse	Nasopharyngeal Salivary	Ct values on RT-PCR	Reduction in virus titre in 2/4 participants for at least 3 h.
[49]	Intervention 5 Control 5	Intervention 32 (13) Control 25 (5)	Intervention 2 Control 5	Y	Oral gargle	Nasopharyngeal Oropharyngeal	Virus clearance on RT-PCR	Comparison of outcomes for the intervention group and the control arm on Day 4, Day 6 and Day 12 showed a significant difference.
[50]	Intervention 11	NA	NA	N	Oral rinse and gargle	Salivary	RNA copies and viral titres on RT-PCR	Viral copies and titres were significantly decreased 60 min after gargling
[51]	Intervention 4 Control 2	Intervention 40.7 (11.5) Control 36 (14.1)	Intervention 4 Control 1	Y	Oral rinse	Salivary	Ct values on RT-PCR	Reduction in virus load was observed 6 h after exposure in the intervention group compared with controls.

### Safety and tolerability of povidone-iodine in the oropharyngeal mucosa

Existing data support the overall safety of povidone-iodine when applied to skin surfaces for wound management and pre-operative skin disinfection protocols [1]. Povidone-iodine (up to a concentration of 10%) has been marketed as a non-prescription medication that is available over-the-counter in many countries (Table 1). At higher concentrations, however, concerns have been raised on the safety of povidone-iodine to skin surfaces, especially to epithelial cells, fibroblasts, granulocytes, monocytes and keratinocytes [55–57]. Under experimental settings, limited data from human epithelial cells suggest potential for cytotoxicity when cells are continuously exposed to high concentrations of povidone-iodine for prolonged periods of up to 24 h [58]. These concerns, however, are not supported by other studies which showed no significant cytotoxicity or delayed wound healing among those who received povidone-iodine at concentrations up to 5% [59,60]. Furthermore, no significant effects on mucociliary function were observed when epithelial cells were exposed to povidone-iodine for 7 weeks, despite lacking the cellular network that protects these cells from cytotoxic injuries [59,61]. *In vivo*, excess amounts of povidone-iodine could possibly be washed away by saliva or redistributed to peripheral and surrounding areas through the mucociliary actions of the cilia. The antimicrobial effects of povidone-iodine appear to spare naturally occurring microbial flora in the oral cavity which may have intracellular niches. One study observed no deleterious effects of povidone-iodine on the nitrate-reducing species (*Veillonella dispar*) following exposure to povidone-iodine [62,63].

#### Irritant and allergenic potential of povidone-iodine

Iodine-containing antiseptics are known to cause skin irritation [64]. Povidone-iodine could trigger a local irritant reaction in areas where there is pooling and prolonged contact with povidone-iodine [64–66], which could be exacerbated by the desiccating effects of alcohol before povidone-iodine use. Compared with other iodine-containing antiseptics (e.g. potassium iodide and alcoholic tincture solutions), the complex of povidone and iodine allows for a gradual release of free iodine which reduces cellular toxicity that might result from a sharp increase in iodine exposure [67]. When applied to a moist surface (e.g. oropharyngeal mucosa), existing data suggest that povidone-iodine may not be as irritating as it is to skin and is generally well-tolerated. In a clinical trial involving 735 participants who received povidone-iodine (0.45% concentration), none reported local irritant effects of povidone-iodine despite applying povidone iodine throat spray 3 times daily to the throat for 42 d [13]. Although largely self-limited nasal tingling has been reported in some studies [44,47,68], data from one clinical trial did not observe alterations in nasal function or detectable damage to the ciliated epithelium [69].

Povidone-iodine is also weakly allergenic. In a patch-test study involving 500 individuals, only 2 (0.4%) participants were found to have a true allergic contact dermatitis reaction to povidone-iodine (1% concentration) [3]. Although anaphylactic reactions have been attributed to povidone-iodine [70–73], questions have been raised about the nature and pathophysiology of these hypersensitivity reaction as some individuals with previous reactions could respond to other allergens present in iodinated radiological contrasts [74,75], or iodine-containing food and medications [76]. Povidone-iodine is not known to stain the teeth [77] and has good acceptability in terms of taste, after-taste, and odour [78]. Rarely, povidone-iodine used to disinfect the oral cavity in preparation for surgery has been reported to cause aspiration pneumonitis following general anaesthesia [79–81].

#### Impact on thyroid function

Small amounts of povidone-iodine may be washed away by saliva and ingested when povidone-iodine is applied to the oral cavity or pharynx. Concerns for the cumulative impact on thyroid function stem from several case reports and smaller cohorts reporting alterations in thyroid hormones following long-term use of povidone-iodine [82–85]. Overt hypothyroidism was reported in an elderly man who gargled 60–90 mL of undiluted povidone-iodine gargle solution (7% concentration) per month for more than 10 years [82], while a cohort analysis of 46 young adults observed mild increase in thyroid-stimulating hormone (TSH) (within limits of normal ranges) after a 6-month use of povidone-iodine gargle (5% concentration) in the treatment of gingivitis and dental plaque [85]. Another study similarly reported a mild increase in serum TSH after povidone-iodine treatment for 24 weeks which normalized after 3 weeks [84]. Among patients diagnosed with SARS-CoV-2 infection, rise in TSH occurred after a 5-d use of povidone-iodine solution (1% concentration), before normalizing after 7–12 d following medication discontinuation [47]. However, one study that applied povidone-iodine gargle (5% concentration) daily for 6 months did not observe significant changes in T3 and T4 levels [85]. Further studies are needed to investigate the risk of mild thyroid dysfunction following povidone-iodine use compared with the benefits in SARS-CoV-2 prophylaxis. Until such safety data become available, certain individuals should be advised to limit or avoid oropharyngeal administration of povidone-iodine, especially those with pre-existing thyroid disease [86], pregnant women [87] and breast-feeding mothers [86].

### Practical considerations when applying povidone-iodine

**Figure 3.**
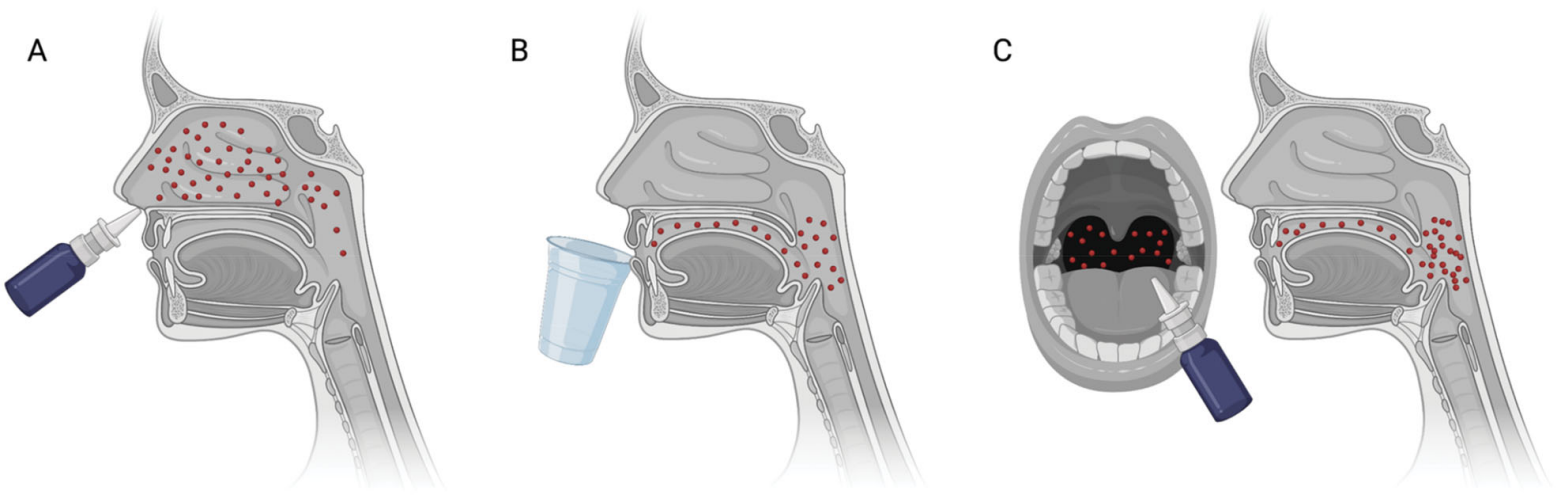
Methods of delivering povidone-iodine to the nasal and oropharyngeal spaces. (A) Nasal drops that are administered through the nostrils could deliver povidone-iodine to the nasal space and, to a lesser extent, the pharynx. (B) Oral gargle solution could cover the oral cavity and pharynx when correctly supervised. (C) Throat spray could direct administration of povidone-iodine to the pharynx. Due to the proximity of the pharynx and oesophagus, occasional swallowing of povidone-iodine could occur with the different methods of delivery.

Unlike the SARS-CoV virus which mostly targets alveolar epithelial cells of the lower respiratory tracts [88,89], the SARS-CoV-2 virus invades and replicates in epithelial cells of the nasal and oropharyngeal cavities [8,90]. Active virus shedding and transmission could last for 10–14 d before acute respiratory symptoms develop [90]. The ability to block virus invasion and create a hostile environment to prevent replication and shedding of virus is believed to explain the role of povidone-iodine. Different methods and modalities of administration have been devised for targeted delivery of povidone-iodine. Nasal drops or spray applications have been administered to target the nasal space, while oral rinse solution mostly covers the oral cavity (Figure 3). Although an oral gargle solution could cover both the oral and pharyngeal spaces, this requires correct gargling techniques, adherence to a minimum gargling duration and need for a sink to expel used solutions [91,92]. Untrained and poorly supervised individuals may instead rinse the oral cavity instead of gargling the solution to cover the pharynx. A study on gargling practices in Japan reported a wide variation in gargling practices which could compromise delivery of povidone-iodine to the intended site of action [93]. Nasal application could trigger one to sneeze while oral gargle could cause aerosolization of virus particles among infected individuals. A directed spray application could perhaps address existing limitations by targeting either the pharynx or nasal space. In the DORM trial, a throat spray was used to deliver povidone-iodine mostly to the pharynx and oral cavity [13]. Although this method of administration does not cover the entire nasal cavity, it is possible that povidone-iodine that is applied to the pharynx could be redistributed to parts of the nasal space through the actions of mucous and cilia, potentially preventing the spread of the SARS-CoV-2 virus into bronchoalveolar tissues. A practical advantage of throat spray is the convenience of timing and tailoring povidone-iodine use to the risk of exposure. In a study involving four patients with SARS-CoV-2 infection, decrease in virus titres was sustained for at least 3 h following povidone-iodine use [48]. Existing data support safety of povidone-iodine when applied topically to the nasal and oropharyngeal mucosa. In an observational study, 0.5% PVP-I gargles and nasal drops have been safely used 3–4 times daily for 5 months [68], whereas 0.45% povidone-iodine was used three times daily for 42 d [13]. To date, the longest duration of povidone-iodine use (1–1.25%) was 28 months, at a frequency of four times daily [94].

### Clinical applications

Transmission of the SARS-CoV-2 is mostly silent [95]. Concerns for uncontrolled transmission have led many countries to impose restrictions on gatherings, wearing of facemasks and limiting travels between countries. Despite mass vaccination, uncontrolled transmission has been reported in wet markets, malls, hospitals, clinics, prisons, household contacts, nursing home facilities and in public transportation (e.g. trains, buses and planes). Uncertainties surrounding the efficacy of vaccination to fully prevent transmission against virus variants and concerns of a waning immunity from vaccination and possibly even prior infection require additional safeguards especially among vulnerable individuals. The ability to tailor povidone-iodine use according to exposure risk is a key advantage of throat application.

Patients with acute SARS-CoV-2 infection harbour a higher virus load and could transmit the virus to their immediate contacts [8]. Among hospitalized patients, the risk of transmission to healthcare workers is especially high during aerosol generating procedures such as endotracheal intubation, and when inserting and manipulating tracheostomy and nasogastric tubes. Suresh and colleagues advocated the use of pre-operative povidone-iodine gargle when preparing patients with COVID-19 undergoing major surgeries [96]. Although it appears intuitive that lowering the amount of live SARS-CoV-2 viruses could reduce the risk aerosolization and the risk of transmitting the virus, data on the efficacy of povidone-iodine to prevent infection in these high-risk settings are lacking. Neither has it been shown that successful lowering of virus load within the nasal nor oropharyngeal cavities could indeed reduce the severity of SARS-CoV-2 infection despite some data linking virus load and disease severity [97].

## Conclusions

Povidone-iodine is a widely available medication that has potent antiviral effects that can be safely administered to the oropharyngeal mucosa to potentially reduce SARS-CoV-2 transmission. Adverse events from povidone-iodine are mostly mild and self-limiting. Although existing data suggest that ingestion of small amounts of povidone-iodine may not affect thyroid function, high-quality data are presently lacking. When applied correctly, povidone-iodine could create a hostile environment for virus replication and alter the transmission dynamics of SARS-CoV-2. Future studies are needed to examine the acceptance and tolerability of povidone-iodine in the community, the role of povidone iodine throat sprays in diverse community and clinical settings and the long-term impact of povidone-iodine use on thyroid function.

## Supplementary Material

Supplemental Material

## Data Availability

The data that support the findings of this study are available in publicly available databases like PubMed, Cochrane, Embase, ClinicalTrials.gov, and World Health Organization’s International Clinical Trials Registry Platform. The search strategy used can be found in Supplementary Table 3.

## Abbreviations

MERS-CoV: Middle East respiratory syndrome–related coronavirus
SARS-CoV: Severe acute respiratory syndrome coronavirus 1
SARS-CoV-2: Severe acute respiratory syndrome coronavirus 2
PCR: Polymerase chain reaction
TSH: Thyroid stimulating hormone
ACE-2: Angiotensin converting enzyme 2
T3: Triiodothyronine
T4: Thyroxine
