# Supplementary material for: Repurposing povidone-iodine to reduce the risk of SARS-CoV-2 infection and transmission: a narrative review
Source: Ann Med. 2022 May 20;54(1):1488–99. doi: 10.1080/07853890.2022.2076902 (PMC9132411; doi:10.1080/07853890.2022.2076902)
Supplement: Supplemental Material [file IANN_A_2076902_SM5162.docx]

**Supplementary Table 1**: PICOS inclusion and exclusion criteria applied to database search

| **PICOS** | **Inclusion Criteria** | **Exclusion Criteria** |
| --- | --- | --- |
| **Population** | - Patients with SARS-CoV-2 infection  - Individuals at high risk for COVID-19 infection due to their comorbidities (eg. immunosuppressed) or due to exposure to high risk situations (eg. healthcare settings, uncontrolled community transmission or crowded settings like dormitories or prisons)  - Individuals of average risk of COVID-19 infection using pre-exposure prophylaxis  - Individuals of average risk of COVID-19 infection using post-exposure prophylaxis  - Animals exposed to SARS-CoV-2  - Cells infected with SARS-CoV-2 | NA |
| **Intervention** | Povidone-iodine in its various formulations (e.g., gargle, surgical scrub, skin cleanser) | NA |
| **Comparison** | Absence of povidone-iodine administration: e.g., no intervention, distilled water, oral Vitamin C | NA |
| **Outcome** | SARS-CoV-2 infection, acute respiratory symptoms, symptomatic infection, pneumonia requiring hospitalisation, SARS-CoV-2 viral load, number of adverse events, time for SARS-CoV-2 viral load reduction, cytotoxic concentration | NA |
| **Study design** | Articles in English or translated to English  All study designs including:   - Randomised controlled trials, cohort studies, case-control studies, cross-sectional studies, descriptive papers, animal studies and in-vitro studies   Year of Publication: Database inception – 2 June 2021  Databases: PubMed, Embase, The Cochrane Library, Clinicaltrials.gov, WHO ICTRP | Ideas, editorials, and perspectives  Reviews and meta-analyses  Clinical trials in progress |

**Supplementary Table 2**: In vitro studies on the use of povidone-iodine against SARS-CoV-1 and MERS-CoV

| ***In vitro* studies** | **SARS-CoV-1 and MERS-CoV** |
| --- | --- |
| Kariwa et al., 2006 | Isodine Scrub (1%), Isodine Palm (0.25%) and Isodine Nodo Fresh (0.23%) reduced SARS-CoV-2 virus titers from 1.17 × 10^6^ TCID50/ml to below the detection limit, <40 to <160, within 1 minute of contact.  However, 1 minute of contact did not reduce viral titers to below detectable levels for Isodine (1%) and Isodine Gargle (0.47%), which had reduction rates of 8.1 × 10−5 and 1.6 × 10−4, respectively. These products required a 2-minute contact time to reduce virus titers to below detectable limits. |
| Eggers et al., 2018 | Application of povidone-iodine gargle/mouthwash diluted 1:10 (equivalent to a concentration of 0.70% povidone-iodine) and 1:30 (equivalent to a concentration of 0.23% povidone-iodine) reduced viral titers of SARS-CoV and MERS-CoV by ≥ 4 log10 within 15 seconds of contact, in both clean and dirty conditions. |
| Eggers et al., 2015 | 4% povidone-iodine skin cleanser and 7.5% povidone-iodine surgical scrub reduced viral titers of MERS-CoV by ≥ 4 log10 within 15 seconds of contact, for both the undiluted and 1:10 dilutions, in both clean and dirty conditions.  1% povidone-iodine gargle/mouthwash, undiluted, demonstrated ≥ 4 log10 reduction in 15 seconds for both clean and dirty conditions. For the 1:10 dilution, 15 seconds of exposure was needed for clean conditions, whereas dirty conditions required 30 seconds. |

**Supplementary Table 3:** Full search strategy for PubMed, Embase and Cochrane

| **PubMed**  ("covid 19"[MeSH Terms] OR "sars cov 2"[MeSH Terms] OR ("covid 19"[Title/Abstract] OR "COVID19"[Title/Abstract] OR "covid 19 virus disease*"[Title/Abstract] OR "covid 19 virus disease*"[Title/Abstract] OR "covid 19 virus infection*"[Title/Abstract] OR "covid 19 virus infection*"[Title/Abstract] OR "2019 ncov infection*"[Title/Abstract] OR "2019 ncov infection*"[Title/Abstract] OR "2019-nCoV Infections"[Title/Abstract] OR "coronavirus disease 19"[Title/Abstract] OR "coronavirus disease 19"[Title/Abstract] OR "2019 Novel Coronavirus Disease"[Title/Abstract] OR "2019 Novel Coronavirus Infection"[Title/Abstract] OR "2019 ncov disease*"[Title/Abstract] OR "2019 ncov disease*"[Title/Abstract] OR "Coronavirus Disease 2019"[Title/Abstract] OR "SARS Coronavirus 2 Infection"[Title/Abstract] OR "sars cov 2 infection"[Title/Abstract] OR "sars cov 2 infection"[Title/Abstract] OR "SARS-CoV-2 Infections"[Title/Abstract] OR "covid 19 pandemic*"[Title/Abstract] OR "covid 19 pandemic*"[Title/Abstract] OR "Wuhan Seafood Market Pneumonia Virus"[Title/Abstract] OR "SARS Coronavirus 2"[Title/Abstract] OR "severe acute respiratory syndrome coronavirus 2"[Title/Abstract] OR "2019 nCoV"[Title/Abstract] OR "2019nCoV"[Title/Abstract] OR "new coronavirus"[Title/Abstract] OR "novel coronavirus"[Title/Abstract] OR "novel corona virus"[Title/Abstract] OR "sars cov 2"[Title/Abstract] OR "2019-novel CoV"[Title/Abstract] OR "ncov19"[Title/Abstract] OR "ncov-19"[Title/Abstract] OR "COVID-19 drug treatment"[Title/Abstract] OR "anti-COVID-19 treatment"[Title/Abstract] OR "COVID-19 diagnostic testing"[Title/Abstract] OR "COVID-19 serotherapy"[Title/Abstract])) AND ("povidone iodine"[MeSH Terms] OR "Chemoprevention"[MeSH Terms] OR ("COVID-19 drug treatment"[Supplementary Concept] AND "povidone iodine"[MeSH Terms]) OR ("Anti-Infective Agents"[MeSH Terms] AND "povidone iodine"[MeSH Terms]) OR ("Antiviral Agents"[MeSH Terms] AND "povidone iodine"[MeSH Terms]) OR ("anti infective agents, local"[MeSH Terms] AND "povidone iodine"[MeSH Terms]) OR ("povidone"[Title/Abstract] OR "chlorhexidine"[Title/Abstract] OR "CHX"[Title/Abstract] OR "PVP"[Title/Abstract] OR "PVP-I"[Title/Abstract] OR "pvp iodine*"[Title/Abstract] OR "Polyvinylpyrrolidone"[Title/Abstract] OR "betadine*"[Title/Abstract] OR "providine*"[Title/Abstract] OR "disadine*"[Title/Abstract] OR "isodine*"[Title/Abstract] OR "pharmadine*"[Title/Abstract] OR "alphadine*"[Title/Abstract] OR "Betaisodona"[Title/Abstract] OR "Tubulicid"[Title/Abstract] OR "Sebidin"[Title/Abstract] OR "MK412A"[Title/Abstract] OR "brauno*"[Title/Abstract] OR "bridin*"[Title/Abstract] OR "Iodopovidone"[Title/Abstract] OR "Povadyne"[Title/Abstract] OR "povidone iodine"[Title/Abstract] OR "Prepodyne"[Title/Abstract] OR "Proviodine"[Title/Abstract] OR "Traumasept"[Title/Abstract] OR "Videne"[Title/Abstract] OR "Chemoprevention"[Title/Abstract] OR "Chemoprophylaxis"[Title/Abstract] OR "Prophylaxis"[Title/Abstract] OR "prophylactic chemotherapy"[Title/Abstract] OR "mass drug administration"[Title/Abstract] OR "post exposure prophylaxis"[Title/Abstract] OR "pre exposure prophylaxis"[Title/Abstract])) |
| --- |
| **Embase**   \| (povidone:ab,ti OR chlorhexidine:ab,ti OR chx:ab,ti OR pvp:ab,ti OR 'pvp i':ab,ti OR 'pvp iodine*':ab,ti OR polyvinylpyrrolidone:ab,ti OR betadine*:ab,ti OR providine*:ab,ti OR disadine*:ab,ti OR isodine*:ab,ti OR pharmadine*:ab,ti OR alphadine*:ab,ti OR betaisodona:ab,ti OR tubulicid:ab,ti OR sebidin:ab,ti OR mk412a:ab,ti OR brauno*:ab,ti OR bridin*:ab,ti OR iodopovidone:ab,ti OR povadyne:ab,ti OR 'povidone iodine':ab,ti OR prepodyne:ab,ti OR proviodine:ab,ti OR traumasept:ab,ti OR 'videne':ab,ti OR chemoprevention:ab,ti OR chemoprophylaxis:ab,ti OR prophylaxis:ab,ti OR 'prophylactic chemotherapy':ab,ti OR 'mass drug administration':ab,ti OR 'post-exposure prophylaxis':ab,ti OR 'pre-exposure prophylaxis':ab,ti OR 'povidone iodine'/exp OR 'chemoprophylaxis'/exp OR ('antiinfective agent'/exp AND 'povidone iodine'/exp) OR ('antivirus agent'/exp AND 'povidone iodine'/exp) OR ('topical antiinfective agent'/exp AND 'povidone iodine'/exp)) AND ('covid 19':ab,ti OR covid19:ab,ti OR 'covid-19 virus disease*':ab,ti OR 'covid 19 virus disease*':ab,ti OR 'covid-19 virus infection*':ab,ti OR 'covid 19 virus infection*':ab,ti OR '2019-ncov infection*':ab,ti OR '2019 ncov infection*':ab,ti OR '2019-ncov infections':ab,ti OR 'coronavirus disease-19':ab,ti OR 'coronavirus disease 19':ab,ti OR '2019 novel coronavirus disease':ab,ti OR '2019 novel coronavirus infection':ab,ti OR '2019-ncov disease*':ab,ti OR '2019 ncov disease*':ab,ti OR 'coronavirus disease 2019':ab,ti OR 'sars coronavirus 2 infection':ab,ti OR 'sars-cov-2 infection':ab,ti OR 'sars cov 2 infection':ab,ti OR 'sars-cov-2 infections':ab,ti OR 'covid-19 pandemic*':ab,ti OR 'covid 19 pandemic*':ab,ti OR 'wuhan seafood market pneumonia virus':ab,ti OR 'sars coronavirus 2':ab,ti OR 'severe acute respiratory syndrome coronavirus 2':ab,ti OR '2019 ncov':ab,ti OR 2019ncov:ab,ti OR 'new coronavirus':ab,ti OR 'novel coronavirus':ab,ti OR 'novel corona virus':ab,ti OR 'sars cov‐2':ab,ti OR '2019‐novel cov':ab,ti OR ncov19:ab,ti OR ncov‐19:ab,ti OR 'covid-19 drug treatment':ab,ti OR 'anti-covid-19 treatment':ab,ti OR 'covid-19 diagnostic testing':ab,ti OR 'covid-19 serotherapy':ab,ti OR 'coronavirus disease 2019'/exp) \|  \| \| --- \| --- \| |
| **Cochrane Library**  ID Search Hits  #1 (povidone or chlorhexidine or CHX or PVP or PVP-I or PVP-iodine* or Polyvinylpyrrolidone or Betadine* or Providine* or Disadine* or Isodine* or Pharmadine* or Alphadine* or Betaisodona or Tubulicid or Sebidin or MK412A OR brauno* OR bridin* OR Iodopovidone OR Povadyne OR Povidone-iodine OR Prepodyne OR Proviodine OR Traumasept OR Videne OR Chemoprevention OR Chemoprophylaxis OR Prophylaxis OR Prophylactic chemotherapy OR Mass drug administration OR post-exposure prophylaxis OR pre-exposure prophylaxis):ti,ab,kw  #2 MeSH descriptor: [Povidone-Iodine] explode all trees  #3 MeSH descriptor: [Chemoprevention] explode all trees  #4 MeSH descriptor: [Anti-Infective Agents] explode all trees  #5 MeSH descriptor: [Antiviral Agents] explode all trees  #6 MeSH descriptor: [Anti-Infective Agents, Local] explode all trees  #7 #4 AND #2  #8 #5 AND #2  #9 #6 AND #2  #10 #1 OR #2 OR #3 OR #7 OR #8 OR #9  #11 MeSH descriptor: [COVID-19] explode all trees  #12 MeSH descriptor: [SARS-CoV-2] explode all trees  #13 (COVID 19 or COVID19 or COVID-19 Virus Disease* or COVID 19 Virus Disease* or COVID-19 Virus Infection* or COVID 19 Virus Infection*):ti,ab,kw  #14 (COVID-19 drug treatment or anti-COVID-19 treatment or COVID-19 diagnostic testing or COVID-19 serotherapy):ti,ab,kw  #15 (Wuhan Seafood Market Pneumonia Virus or SARS Coronavirus 2 or severe acute respiratory syndrome coronavirus 2 or 2019 nCoV or 2019nCoV or new coronavirus or novel coronavirus or novel corona virus or SARS CoV‐2 or 2019‐novel CoV or ncov19 or ncov‐19):ti,ab,kw  #16 (2019 nCoV Disease* or Coronavirus Disease 2019 or SARS Coronavirus 2 Infection or SARS-CoV-2 Infection or SARS CoV 2 Infection or SARS-CoV-2 Infections or COVID-19 Pandemic* or COVID 19 Pandemic*):ti,ab,kw  #17 (COVID 19 or COVID19 or COVID-19 Virus Disease* or COVID 19 Virus Disease* or COVID-19 Virus Infection* or COVID 19 Virus Infection* or Coronavirus Disease-19 or Coronavirus Disease 19):ti,ab,kw  #18 #11 OR #12 OR #13 OR #14 OR #15 OR #16 OR #17  #19 #10 AND #18 |
